# Spermatic Cord Walthard Cyst as Inguinal Mass: CT/MRI and Immunohistochemical Correlation

**DOI:** 10.70352/scrj.cr.25-0625

**Published:** 2025-12-13

**Authors:** Rintaro Sakamoto, Hiroyuki Tomita, Shota Nakashima, Kimihiro Hattori, Machi Mizuno, Takuji Sakuratani, Hiroomi Ikesyoji, Noriaki Kojima, Kimitoshi Nishio, Tatsumi Iida, Kota Sakurai, Takao Takahashi

**Affiliations:** 1 Department of Surgery, Gifu Seino Medical Center, Seino Kousei Hospital, Ibi-gun, Gifu, Japan; 2 Division of Pathology, Gifu Seino Medical Center, Seino Kousei Hospital, Ibi-gun, Gifu, Japan; 3 Department of Tumor Pathology, Gifu University Graduate School of Medicine, Gifu, Gifu, Japan; 4 Department of Radiology, Gifu Seino Medical Center, Seino Kousei Hospital, Ibi-gun, Gifu, Japan

**Keywords:** Walthard cyst, Walthard cell nests, inguinal canal, spermatic cord, cystic lesions, immunochemistry, transitional epithelium, ultrasonography, CT, MRI

## Abstract

**INTRODUCTION:**

Walthard cell nests are benign urothelial-like rests whose cystic transformation results in Walthard cysts. Their occurrence in male paratesticular structures is rare and can mimic urothelial or mesothelial disease.

**CASE PRESENTATION:**

An 85-year-old male with a history of left inguinal hernia repair presented with a 33-mm whitish cyst adherent to the hernia sac. Ultrasonography revealed a well-defined, hypoechoic mass without internal vascular flow. Non-contrast CT revealed a lobulated, hyperattenuating lesion (~196 HU); in contrast, MRI showed intermediate-to-slightly high T1 and low T2 signals without diffusion restriction, consistent with a protein-rich cyst. Intraoperatively, the cyst ruptured, releasing whitish turbid fluid, and was excised en bloc during mesh plug repair. Cytology revealed crystalline material without cells, and cultures were negative. Histologic examination showed a thin-walled fibrous cyst lined by multilayered transitional-type epithelium without atypia. Immunohistochemistry demonstrated positivity for CK5/6, CK7, GATA3, p63, and WT1; negativity for CK20, uroplakin III, PAX8, calretinin, and OCT3/4; and a Ki-67 index of <1%.

**CONCLUSIONS:**

This case represents the 2nd full-length, peer-reviewed report of a spermatic cord/inguinal canal Walthard cyst of this size. Compared with previous reports, our case uniquely offers comprehensive CT/MRI correlation, a complete immunophenotypic profile—including WT1 positivity with CK20 and uroplakin III negativity—and precise size documentation, thereby aiding in the prevention of misdiagnosis and overtreatment of cystic lesions in the inguinal canal of males.

## Abbreviations

BMI
: body mass index
EHS
: European Hernia Society

## INTRODUCTION

Walthard cysts, first described in 1903, are benign epithelial lesions exhibiting transitional or urothelial-type differentiation.^1)^ They are most commonly identified incidentally in gynecologic specimens, particularly within the fallopian tube subserosa, mesosalpinx/mesovarium, and ovarian hilum.^2,3)^ Their occurrence in males is exceedingly rare, with only isolated reports in the epididymis, tunica albuginea, testes, and spermatic cord.^1,2,4)^ Despite their benign nature, these lesions may mimic metastatic urothelial carcinoma or mesothelial proliferations both histologically and immunohistochemically, posing a diagnostic challenge. Accurate diagnosis relies on morphology supported by a characteristic immunophenotype—positivity for CK7, GATA3, p63, and CK5/6, and negativity for CK20, uroplakin III, PAX8, and calretinin.^1–3)^ Here, we report a case of a Walthard cyst arising in the spermatic cord discovered during inguinal hernia surgery. To our knowledge, this is only the third reported case worldwide and the second to include detailed clinicopathological characterization.^1)^ This statement is based on a comprehensive literature search conducted in PubMed and Google Scholar up to September 2025 using the search terms (“Walthard cysts” OR “Walthard cell nests” OR “Walthard rests”) AND (“spermatic cord” OR “inguinal canal”). No additional full-length, peer-reviewed case reports describing Walthard cysts arising in the spermatic cord or inguinal canal of males were identified. Increased recognition of this rare benign lesion may help prevent misdiagnosis as a malignant or metastatic tumor and thus avoid unnecessary procedures such as radical orchiectomy or extensive dissection. Furthermore, this case provides valuable radiologic–pathologic correlation that may assist clinicians and radiologists in the differential diagnosis of cystic lesions within the inguinal canal.

## CASE PRESENTATION

An 85-year-old male presented with a swelling in the left inguinal region that had progressively enlarged. He initially consulted a local physician, was diagnosed with an inguinal hernia, and was subsequently referred to our hospital. His medical history included a pelvic ring fracture treated with transcatheter arterial embolization one year earlier, concomitant fractures of the left atlas and C7 vertebra managed conservatively, and a cerebellar infarction during recovery from the pelvic fracture. On examination, he measured 160 cm in height and weighed 55 kg (BMI, 21.4 kg/m^2^). A soft, elastic, quail egg–sized mass was palpable in the left inguinal region. Laboratory tests revealed no abnormalities, and tumor markers were not assessed.

### Investigations

**Fig. 1:**
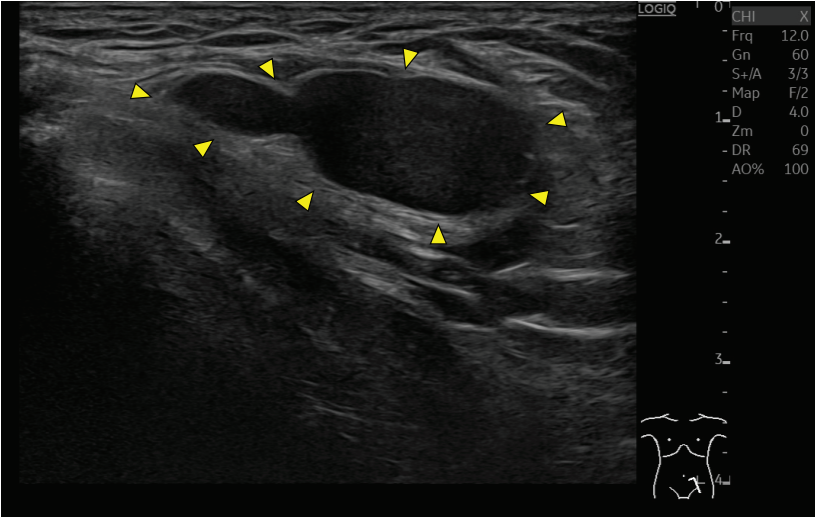
Ultrasonographic findings. A 28 × 11 × 33-mm hypoechoic mass is seen in the subcutaneous fat layer of the left groin (arrowheads). The lesion shows slightly heterogeneous internal echoes, smooth margins, mild posterior acoustic enhancement, and no connection with the epidermis.

**Fig. 2:**
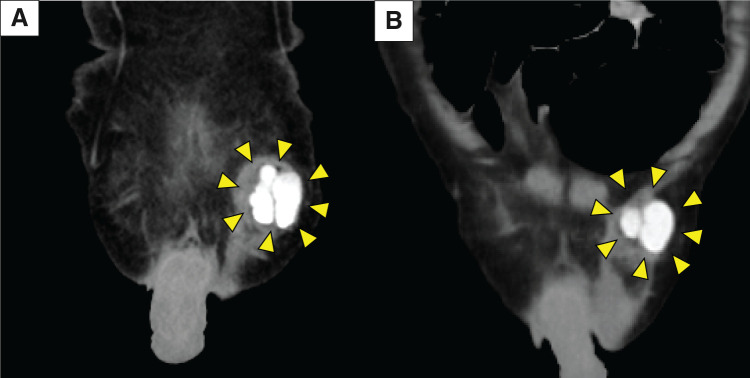
CT findings. (**A**) A lobulated, hyperdense lesion (maximum diameter, 31 mm; attenuation, 196 HU) is present in the left inguinal canal (arrowheads). (**B**) One year earlier, a similar lesion measuring 26 mm had been detected (arrowheads).

**Fig. 3:**
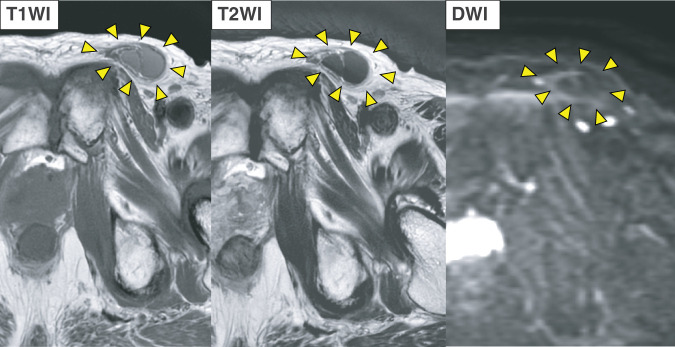
MRI findings. A lobulated 33 mm mass at the entrance of the inguinal canal is visualized (arrowhead). The lesion demonstrates low signal intensity on T2-weighted imaging, intermediate to mildly high signal intensity on T1-weighted imaging, and no restricted diffusion on diffusion-weighted imaging. DWI, diffusion-weighted imaging; T1WI, T1-weighted imaging; T2WI, T1-weighted imaging

Ultrasonography revealed a 28 × 11 × 33-mm hypoechoic mass within the subcutaneous fat layer of the left groin, characterized by slightly heterogeneous internal echoes, smooth margins, mild posterior acoustic enhancement, and no internal blood flow (**Fig. 1**). No communication with the skin surface was observed. CT revealed a lobulated, hyperdense lesion (maximum diameter, 31 mm; attenuation, 196 HU) within the left inguinal canal; a similar lesion measuring 26 mm had been noted one year earlier (**Fig. 2**). MRI showed a lobulated 33 mm mass at the entrance of the inguinal canal with low signal intensity on T2-weighted images, intermediate to mildly high signal intensity on T1-weighted images, and no restricted diffusion on diffusion-weighted images (**Fig. 3**).

### Treatment/surgical findings

**Fig. 4:**
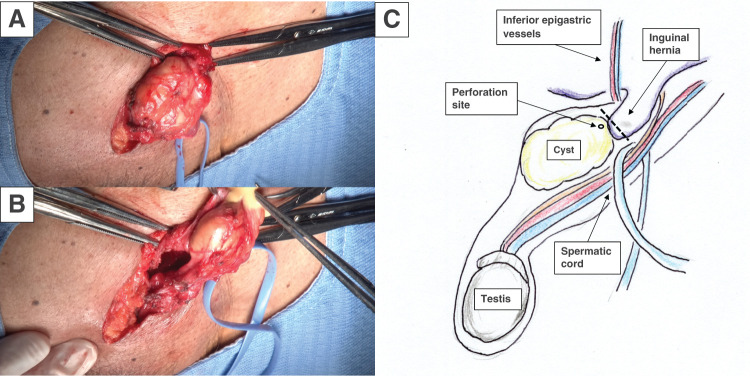
Intraoperative findings. (**A**) A soft, whitish mass was identified in the inguinal canal. (**B**) The cyst ruptured intraoperatively, releasing whitish, turbid, viscous fluid. (**C**) Schematic illustration of the surgical findings showing the hernial sac (broken line) attached to the tumor.

Under spinal anesthesia, an inguinal incision was made. A soft, whitish mass was identified within the inguinal canal and carefully dissected. An indirect inguinal hernia was also noted, with the mass firmly adherent to the hernia sac. The mass was excised along with a portion of the hernia sac. During the procedure, the cyst ruptured, releasing whitish, turbid, viscous fluid (**Fig. 4**). After rupture of the cyst, the operative field was thoroughly irrigated with sterile saline. The hernia orifice measured approximately 2 cm, corresponding to European Hernia Society (EHS) classification L2.^5)^ Hernia repair was subsequently performed using the mesh-plug technique. The operative time was 92 min, and blood loss was 16 mL.

### Pathological findings

**Fig. 5:**
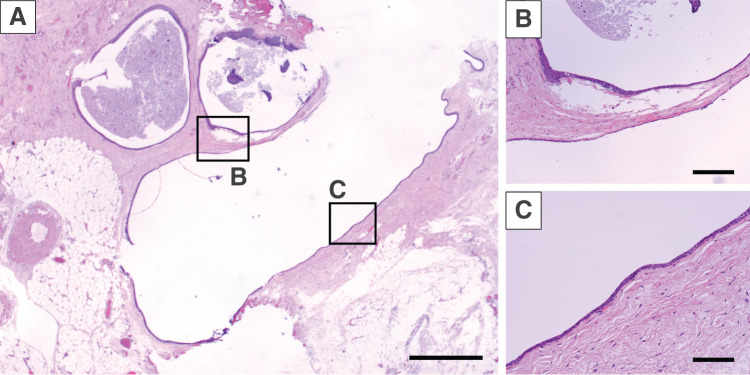
Histopathology of the cyst (H&E). (**A**) Low-power view of a thin-walled cyst adjacent to the inguinal hernia sac. (**B**) The cyst wall consists of a delicate fibrous capsule lined by multilayered cuboidal-to-polygonal epithelium with umbrella cell–like features. (**C**) High-power view demonstrating bland nuclei without atypia, mitotic figures, or necrosis. Scale bars: (**A**) 500 μm; (**B**, **C**) 100 μm. H&E, hematoxylin and eosin

**Fig. 6:**
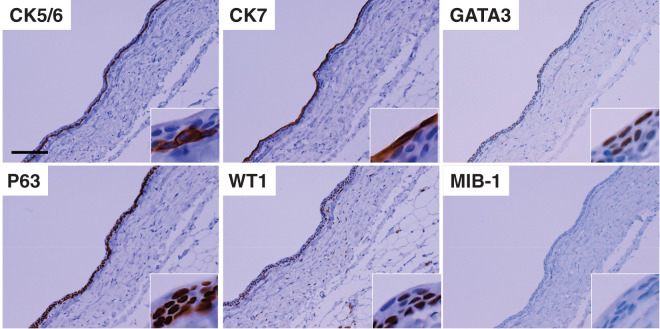
Immunohistochemical profile of the cyst lining. Diffuse cytoplasmic/membranous staining for CK5/6 and CK7 and nuclear positivity for GATA3, p63, and WT1 are demonstrated. The MIB-1 (Ki-67) index is <1%. The consolidated immunophenotype: CK5/6, CK7, GATA3, p63/WT1 positive; CK20, uroplakin III, PAX8, calretinin, OCT3/4 negative (negatives not shown). This profile supports a benign Walthard cyst and excludes urothelial carcinoma and mesothelial lesions. Insets show high-magnification images. Scale bars: 100 μm.

Bacterial culture of the cystic fluid yielded no growth, and cytologic examination revealed crystalline material without cellular elements. Histologically, the lesion was a thin-walled cyst with a fibrous capsule lined by multilayered cuboidal to polygonal epithelial cells displaying bland nuclear features and umbrella cell–like morphology (**Fig. 5**). No cytologic atypia, mitotic figures, or necrosis were identified. Immunohistochemically, the epithelial lining was positive for CK5/6, CK7, GATA3 (**Fig. 6**), and p63; however, it was negative for CK20, uroplakin III, calretinin, OCT3/4, and PAX8. The MIB-1 (Ki-67) index was <1%. These findings were consistent with a benign Walthard cyst.

### Outcome and follow-up

The postoperative course was uneventful, and the patient was discharged on POD 2. No recurrence was observed at one month postoperatively.

## DISCUSSION

Walthard cell nests are benign epithelial proliferations exhibiting transitional (urothelial-like) differentiation. When cystic change occurs, the lesion is termed a Walthard cyst.^1,2)^

Walthard cell nests are clusters of transitional-type epithelial cells most often identified incidentally in gynecologic sites such as the fallopian tubes, ovaries, and broad ligaments.^2)^ Although typically asymptomatic, they have also been reported in extragynecologic locations—including the inguinal canal, intestines, appendix, and mesentery—indicating that their distribution is not confined to the female reproductive tract.^1,2,4,6,7)^ Notably, lesions arising in the inguinal canal are consistently cystic and relatively large, with features distinct from those seen at other sites. Reports involving the inguinal canal or spermatic cord are exceedingly rare, with only two cases described previously; thus, the present case represents the third such report worldwide (**Table 1**).

**Table 1:** Reported cases of Walthard cell rests in the inguinal canal or spermatic cord

Author/year	Age/sex	Location	Size	Imaging modality	Immunohistochemistry
Abdelmalak et al./2018	55/M	Spermatic cord	20 × 10 × 19 mm	Incidental (surgery)	CK5/6/7/GATA3 (+), SALL4, and PAX8 (–)
Fahim et al./2022	80s/M	Right spermatic cord (inguinal hernia)	24 × 10 × 5 mm	US: hernia sac only	CK5/CK7/GATA3 (+), CK20/PAX8/calretinin/OCT3/4 (–)
Present case/2025	85/M	Left inguinal canal (with hernia sac)	28 × 11 × 33 mm	US: hypoechoic mass	CK5/6/CK7/GATA3/p63/WT1 (+), CK20/uroplakin III/PAX8/calretinin/OCT3/4 (–), Ki-67 (MIB-1) <1%
				CT: 196 HU lobulated lesion	
				MRI: T2 low, T1 iso-to-slightly high, DWI negative	

DWI, diffusion-weighted imaging; M, male; US, ultrasound

To our knowledge, this is the 1st documented Walthard cyst with detailed CT and MRI findings. The lesion arose in the left inguinal canal, and preoperative imaging made the differential diagnosis challenging. Noncontrast CT revealed high attenuation (196 HU). In contrast, MRI showed mild-to-moderate hyperintensity on T1-weighted images, hypointensity on T2-weighted images, and no diffusion restriction. Ultrasonography revealed a hypoechoic mass with slightly heterogeneous internal echoes, smooth margins, mild posterior acoustic enhancement, and no vascular flow or communication with the epidermis. Surgical and histopathological examination of the excised specimen confirmed a cystic lesion containing viscous material.

The differential diagnosis for cystic lesions in the inguinal canal includes spermatic cord hydrocele, epidermoid cyst, hematoma, and abscess. Ultrasonography is particularly useful for distinguishing cystic from solid lesions.^8)^ On CT, high-attenuation cysts generally differ from simple cysts, reflecting hemorrhagic or proteinaceous content.^9,10)^ On MRI, cystic lesions with high T1 and low T2 signals are often associated with subacute hematomas or protein-rich cysts.^10,11)^ In this case, the absence of diffusion restriction on diffusion-weighted imaging effectively excluded abscesses and epidermoid cysts. A subacute hematoma secondary to a prior pelvic fracture was initially suspected; however, this was ruled out because the lesion had already been present on earlier CT imaging. Intraoperatively, the cyst contained viscous fluid without bacterial growth or cellular elements, leading to the final diagnosis of a protein-rich cyst.

The key features of this case are as follows: (i) although CT and MRI suggested a hemorrhagic lesion, it was ultimately a protein-rich cyst, and (ii) ultrasonography revealed typical cystic characteristics, including smooth margins, absent vascularity, and posterior acoustic enhancement. These findings suggest that protein concentration and viscosity can significantly influence the imaging appearance of cystic lesions. Although it remains uncertain whether all Walthard cysts share similar features, the findings in this case support including Walthard cysts in the differential diagnosis of cystic inguinal masses.

Walthard cysts are benign, and complete excision is considered curative; however, preoperative diagnosis can be challenging. In cases of progressive enlargement, as in the present case, malignancy could not be definitively excluded, and surgical excision serves both diagnostic and therapeutic purposes. Walthard cysts in males are exceedingly rare, underscoring the importance of recognizing this entity to avoid misdiagnosis and unnecessary aggressive treatment.

In this case, the intraoperative measurement of the hernia orifice was approximately 2 cm, corresponding to EHS classification L2. Given this defect size, mesh repair is generally recommended by current international guidelines to reduce the risk of recurrence.^5)^ In addition, although the presence of the mass within the inguinal canal may have masked clinical signs of herniation, mesh repair was selected because removal of the mass was expected to unmask the underlying hernia and potentially cause postoperative symptoms. Although the cyst ruptured during dissection, the operative field was thoroughly irrigated, no contamination was observed, and bacterial cultures yielded no growth; therefore, mesh placement was considered appropriate. The cyst wall was completely excised, and cytologic analysis of the cystic fluid revealed no cellular components. Given that a Walthard cyst is a benign epithelial lesion, the risk of recurrence after rupture is considered to be minimal.

Histologically, the cyst demonstrated a fibrous wall lined with multilayered cuboidal to polygonal cells exhibiting umbrella cell–like features, without atypia, mitoses, or necrosis. The immunophenotype—positivity for CK5/6, CK7, GATA3, and p63, and negativity for CK20, uroplakin III, PAX8, OCT3/4, and calretinin, with a Ki-67 index <1%—is diagnostic of a benign Walthard cyst.^1,3)^ This immunoprofile is essential for excluding mimics such as metastatic urothelial carcinoma and mesothelial lesions.

This report has several limitations. First, it presents a single rare case, and the imaging findings and pathological features cannot be generalized to all Walthard cysts. Second, the follow-up period was relatively short; although the likelihood of recurrence is considered low, the long-term prognosis remains uncertain. Finally, the pathophysiology underlying cyst formation within Walthard cell nests is still unclear, and further case accumulation is needed to clarify these issues.

## CONCLUSIONS

In summary, this case represents the third reported instance of a Walthard cyst arising in the inguinal canal. It is notable for being the largest such lesion reported to date and the first to include detailed CT and MRI findings. Moreover, the incorporation of novel immunohistochemical results further strengthens its clinical and pathological relevance. This report underscores the importance of including Walthard cysts in the differential diagnosis of inguinal masses.

## DECLARATIONS

### Availability of data and materials

All data generated or analyzed during this study are included in this published article.
